# Phytoremediation Potential of Macrophytes of Urban Waterbodies in Central India

**DOI:** 10.5696/2156-9614-9.24.191206

**Published:** 2019-11-27

**Authors:** Sandeep K. Pandey, Ritambhara K. Upadhyay, Vineet Kumar Gupta, Kenate Worku, Dheeraj Lamba

**Affiliations:** 1 International Water Management Institute, New Delhi, India; 2 Institute of Technology, Jimma University, Jimma, Ethiopia; 3 Larsen & Toubro, Lucknow, India; 4 College of Social Sciences and Humanities, Jimma University, Jimma, Ethiopia; 5 Institute of Health, Jimma University, Jimma, Ethiopia

**Keywords:** urban lake, domestic sewage pollution, natural vegetation, bioaccumulation, translocation of metals

## Abstract

**Background.:**

India's rapidly increasing population and growing urbanization pose a great challenge for wastewater treatment efforts, leading to increased pollution of surrounding waterbodies.

**Objectives.:**

A field sampling-based study was conducted to analyze water quality, heavy metals and bioconcentration and bioaccumulation in the roots and shoots of naturally growing vegetation in an urban lake, Laxmi Taal. The lake receives domestic sewage from Jhansi city in Central India.

**Methods.:**

Temperature, pH, electrical conductivity, turbidity, and water-soluble ions were measured with appropriate instruments. Plant accumulation of metals was measured with the bioconcentration factor (BCF), the ratio of metal concentration in the root to wastewater. The translocation factor (TF) was estimated as the ratio of metal concentration in the shoot to the root.

**Results.:**

Water quality and heavy metal concentrations were found to be within the prescribed limit as per Indian standards IS-2296 “D”. In the present study, BCF was assessed to be >1 and the plants Typha angustifolia and Echhornia crassipus were determined to be accumulator plants. The TF study revealed that translocation of all the metals studied were significant, except for manganese (Mn), where concentration was found to be below detection limit.

**Conclusions.:**

The present study validated that Typha angustifolia and Echhornia crassipus could be used for bioremediation purposes in cases of urban waterbodies receiving varying amounts of domestic wastewaters which have relatively limited concentrations of toxic metals.

**Competing Interests.:**

The authors declare no competing financial interests

## Introduction

With increasing population and growing urbanization in India, wastewater generation is also increasing at a rapid pace. Lack of appropriate treatment facilities and inadequate policy planning at the local level has aggravated the issue and many rivers, lakes, wetlands, lakes and other waterbodies are polluted.^1^,^2^ Increased pollutants in waterways causes cultural eutrophication.^3^ Untreated municipal and partially treated sewage and other external inputs causes changes in surface water quality.^4^ Municipal sewage contains partially decomposed materials (inorganic and organics) and trace elements including cadmium (Cd), chromium (Cr), nickel (Ni), lead (Pb), copper (Cu), zinc (Zn), manganese (Mn) and iron (Fe)*.* Waterbodies consisting of turbid heterogeneous liquid with persistent chemicals pose serious challenges due to water-borne diseases and health hazards.^5–7^

Laxmi Taal in Jhansi, Bundelkhand (Uttar Pradesh) is a historical, previously rain-fed lake of about 32.52 hectares. Today, Laxmi Taal is encroached with an inflow of urban sewage, principally carrying domestic wastewater and runoff from surrounding settlements, temples, gardens and farmland without undergoing any treatment or sedimentation. Monitoring and assessment of water contamination, especially heavy metal accumulation, is a serious concern, as inhabitants depend on the lake for vegetable irrigation, drinking water for livestock and recreation. The consequences of pollution on plants and animals has been extensively studied in lentic and lotic ecosystems.

Phytoremediation can be used as an alternative solution for heavy metal remediation processes due to advantages as a cost-effective, efficient, environment- and eco-friendly technology based on the use of metal-accumulating plants.^8^,^9^ Acidic water aids the uptake of heavy metals by plants and the enrichment mechanisms are related to the surface area of the plant exposed to water.^10^ Eucalyptus camaldulensis, Zea mays, Potamogeton pectinatus L., and Typha domingensis are some of the best candidate species as hyperaccumulators for phytoremediation of heavy-metal contaminated soils and water.^11–15^ To appropriately plan remedial measures using vegetation for heavy metal removal, it is imperative to have adequate knowledge of the role played by detritus (sink/source).^16^

Phytoremediation concerns have gained considerable attention in warm sub-tropical to semi-arid regions. In water scarce areas, surface waterbodies are the main source of water for ‘B’ grade use. The present study aims to provide an assessment of water quality, level of heavy metals and a comparative phytoremediation potential of Typha angustifolia and Echhornia crassipus. These two species are commonly occurring pre-adopted, successful metal accumulators.^17^,^18^ An extensive study was carried out on their phytoremediation potential in high metal contamination areas. The present study assesses the metal accumulation potential of the species with lower concentrations of heavy metal contamination. The outcome of the study may be useful in planning for bioremediation and restoration measures for urban waterbodies.

## Methods

Laxmi Taal is a shallow, fresh water urban lake in the city of Jhansi, spreading over an area of about 0.162 km^2^. Laxmi Taal is located between latitude 25°27′20″- 25°27′50″N and longitude 78°35′20″- 78°35′45″E. With temples all along its boundary, it is a central part of the historical, cultural and recreational life of Jhansi city. It is approximately 32.52 hectares across with an average depth of 2.5 m and has a catchment area of 2370 hectares. The sewage carrying domestic wastewater of Jhansi city is dumped into Laxmi Taal through various *nallas* (channels), namely Kuberau nala, Kasai mandi nala, Laxmi gate nala, Jashiyana nala, Banglaghat nala and Bludgeon nala. A large influx of people in the last few decades around the fringe of the lake has resulted in rapid deterioration of water quality. Increased inflow of untreated sewage, disposal of municipal solid wastes, excessive use of fertilizers and pesticides are some of the major problems facing the lake environment.

**Abbreviations**
*BCF*: Bioconcentration factor
*DO*: Dissolved oxygen
*TF*: Translocation factor

### Water sample collection and analysis

**Figure 1:**
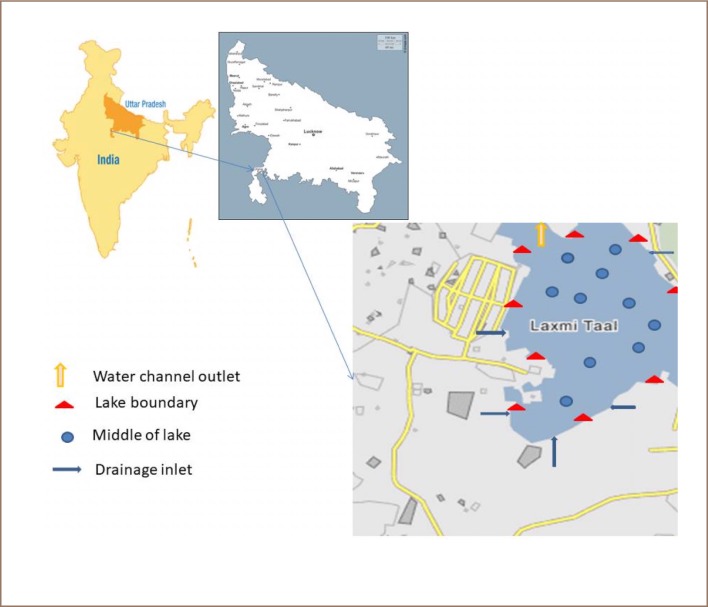
Map showing the study area and sampling site in Laxmi Taal

The lake water samples were collected in precleaned, acid-treated high-density 3L polythene bottles, in triplicate at approximately 30-day intervals from each sampling location in the study area. The sampling area was divided into four zones: inlet lake interface, depicted as blue arrows in Figure 1; lake boundary with macrophytes, depicted as red triangles; middle of lake, depicted by a blue circle; and lake outlet, depicted with a yellow arrow. The four water sampling sites were selected on the basis of input, regeneration capacity, open area and outlet. Water parameters were determined in the present study and the methods adopted are described below. The water samples were brought to the Institute of Environment and Development Studies, Bundelkhand University, Jhansi for analysis. Each group of samples was analyzed separately. The analysis work was carried out according to the procedures described below.

Temperature was measured with the help of thermometer, pH by a pH meter (Systronics, MK-VI), electrical conductivity (μscm^−1^) by conductivity meter (Systronic, Serial No. 13613), turbidity (nephelometric turbidity units) was determined by a turbidity meter, water soluble ions (sodium (Na^+^) and potassium (K^+^) in the water sample were determined with the help of a flame photometer (Systronics-350), and calcium (Ca^+^) was determined by the versenate titration method using ethylenediaminetetraacetic-disodium salt solution as chelate). Additionally, magnesium (Mg^+^) in ppm, free carbon dioxide (CO_2_), dissolve oxygen (mg L^−1^), chemical oxygen demand (mg L^−1^), carbonate (CO_3−_ mg L^−1^), bicarbonate (HCO_3−_ mg L^−1^) and all other parameters were calculated by standard methods.^19^,^20^

### Plant sampling

A total of 30 samples of T. angustifolia and E. crassipus plants were collected from the study area and their root and shoot system separated. Plant tissue samples were thoroughly washed with running tap water and rinsed with deionized water to remove any sediment particles attached to the plant surfaces. Plant tissue samples were oven dried at 70°C to constant weight, then the dried materials were ground into powder and preserved in paper bags in desiccators for subsequent analysis. The samples were digested following the procedure described by the AOAC official method 985.01.^21^

Plant accumulation of metals was measured with the bioconcentration factor (BCF), the ratio of metal concentration in the root to wastewater. Translocation factor (TF) was estimated as the ratio of metal concentration in the shoot to the root.^22^

## Results

Analysis of pH is an important parameter in an examination of water quality. In the present study, the pH value varied between 7.463 and 7.743 during the summer season. It was found to be close to the prescribed limit of surface water quality category-A (pH 8.5). Turbidity ranged from 16.10 to 46.183 (nephelometric turbidity units). Turbidity is the measurement of scattered light at 90° in a water system and can be a hindrance to submerged plant growth.

Conductivity (μS cm^−1^) varied from 1399.66 to 1751.16 in Laxmi Taal during the summer season *(Table 1)*. Conductivity of water varies directly with the temperature and is proportional to dissolved mineral matter content. Dissolved oxygen (DO) is the most critical factor in determining water quality and reflects the physical and biological process prevailing in the water (nutrient load). In Laxmi Taal lake, the DO varied from 3.18 to 4.62 mg L^−1^ during the summer season. The high DO content in the surface water may be a result of direct contact of the surface water layer which enhances the dissolution of oxygen in water. A minimum of 4 mg/l of DO should be maintained in water for healthy growth of biota.^23^ Chemical oxygen demand in Laxmi Taal was found in the range of 110.66 to 179.00 mg L^−1^ during the summer season. The chemical oxygen demand was high at the drainage inlet due to the demand of oxygen from the degradation of waste.

**Table 1:** Water Quality Results from Laxmi Taal

**Parameters**	**Sites under study**				Surface water quality standard: IS:2296^*^^24^
	**Lake boundary**	**Middle of lake**	**Drainage inlet**	**Water channel outlet**	

Temperature	16.40	16.50	16.30	16.46	-
Turbidity	46.18	16.10	17.90	22.50	-
pH	7.64	7.74	7.743	7.46	8.5
Electrical conductivity	1751.16	1704.00	1547.33	1399.66	-
Total solids	1031.40	756.05	697.63	745.33	-
Total dissolved solids	863.66	610.85	635.66	655.66	500
Total suspended solids	167.73	145.20	60.53	88.00	-
Dissolved oxygen	4.56	3.184	4.62	4.37	6
Biochemical oxygen demand	82.55	57.81	75.35	59.77	2
Chemical oxygen demand	179.00	159.71	124.33	110.66	
Hardness total	216.00	199.71	186.67	288.67	300
Calcium hardness	119.70	133.10	110.43	99.60	-
Magnesium hardness	96.30	86.61	75.77	188.17	-
Free CO_2_	79.20	52.49	71.60	73.73	-
Carbonate	81.62	76.26	77.25	74.00	-
Bicarbonate	115.19	114.73	135.46	128.14	-
Sodium	253.47	241.40	232.25	229.47	-
Potassium	58.52	50.44	41.55	40.70	-
Calcium	47.94	45.30	46.76	41.74	80
Magnesium	23.41	21.06	18.98	22.55	24
Chloride	210.16	223.75	180.57	243.34	250

^*^ Drinking water without conventional treatment but after disinfection (category-A).

**Table 2:** Heavy Metal Content in Laxmi Taal Across Study Sites

	**Heavy metals under study μg/l**					

	**Mn**	**Cu**	**Zn**	**Ni**	**Pb**	**Fe**
**Surface water quality standard IS:2296 Study Sites**	500	1500	15000	3000^	100	300
Boundary	BDL	23.87±3.5	269.70±49.84	18.50±6.10	114.62±17.60	1089.62±44.94
Middle	BDL	101.37±4.5	259.49±25.70	7.00±1.90	28.50±7.90	1026.87±24.95
Inlet	BDL	24.12±2.16	297.35±95.62	7.00±1.80	18.00±5.60	762.75±18.87
Boundary	BDL	141.75±8.2	455.50±61.98	44.37±4.10	40.87±6.00	1107.00±11.54
Middle	BDL	23.87±3.5	269.70±49.84	18.50±6.10	114.62±17.60	1089.62±44.94
Outlet	BDL	101.37±4.5	259.49±25.70	7.00±1.90	28.50±7.90	1026.87±24.95

Abbreviation: BDL, below detection limit; Cu, copper; Fe, iron; Mn, manganese; Ni, nickel; Pb, lead; Zn, zinc.

**Table 3:** Heavy Metal Content (μg/l) in Typha angustifolia and Echhornia crassipus Grown in Laxmi Taal

**Name of the plant**	**Plant parts**	**Mn**	**Cu**	**Zn**	**Ni**	**Pb**	**Fe**
T. angustifolia	Root	52.729±13.82	4.284±0.62	26.035±4.63	9.947±2.90	6.056±1.01	194.076±30.45
	Shoot	934.339±105.23	8.984±1.69	97.580±5.22	7.713±0.61	29.474±3.37	1601.470±568.27
E. crassipus	Root	188.680±26.40	7.800±3.57	60.380±2.43	3.080±1.43	12.880±0.94	834.000±124.36
	Shoot	854.250±316.28	62.500±17.53	238.100±76.36	12.850±8.42	25.150±1.20	946.400±459.34

Abbreviation: Cu, copper; Fe, iron; Mn, manganese; Ni, nickel; Pb, lead; Zn, zinc.

**Table 4:** Bioconcentration Factor and Translocation Factor of Heavy Metals

	**BCF shoot/concentration in lake water**	**BCF root/concentration in lake water**	**TF shoot/root**	**BCF shoot/concentration in lake water**	**BCF root/concentration in lake water**	**TF shoot/root**

**Metals**	**Typha angustifolia**			**Eichhornia crassipus**		
Mn	-	-	0.00	-	-	0.00
Cu	0.08	0.04	2.00	0.61	0.12	5.08
Zn	0.37	0.10	3.70	0.91	0.25	3.64
Ni	1.42	1.1	1.29	1.83	0.24	7.63
Pb	1.03	0.21	4.90	0.88	0.51	1.73
Fe	1.56	0.18	8.67	0.92	0.88	1.045

Abbreviation: BCF, bioconcentration factor; Cu, copper; Fe, iron; Mn, manganese; Ni, nickel; Pb, lead; TF, translocation factor; Zn, zinc.

Levels of free CO_2_ were found to be 79.20, 52.49, 71.60, and 73.73 at sampling locations 1–4, respectively. These values were higher than the national surface water quality grade-D standard in India. This indicates intense oxidative activities in the water, which increases the level of free CO_2_, similar to the levels of bicarbonate (114.73, 135.46, 115.19, 128.14) and carbonate levels (76.26, 77.25, 81.62 and 74.00). Levels of carbon in this lake were found to be unusually high.

Total solids reflect the suspended and dissolved materials in inorganic and organic forms. The levels in the form of total solids, total dissolved solids and total suspended solids were collected. The total solid levels were 756.05, 697.63, 1031.40 and 745.33 mg/l. The increased levels in the present study may be due to increased mineralization and waste disposal activities. In this reflection, the concentrations of sodium, potassium, calcium, magnesium and chloride were at significant levels as shown in Table 1.

The BCF or enrichment coefficient is the ratio of concentration of element present in the surroundings to the plant tissue at the time of harvest as characterized by Equation 1.

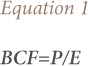
where, P represents the trace element concentration in plant tissues (mg/kg dry wt) and E represents the trace element concentration in the water (mg/l). Higher BCF values indicate higher accumulations in plants.

All the sampling values of heavy metals were lower than the prescribed values for surface water quality (IS: 2296). The luxuriant growth of Typha angustifolia indicated favorable conditions. The enrichment coefficient study and translocation factor studies revealed that the bioconcentration factor was >1 in all of the cases. An enrichment coefficient of >1 indicates that the plant is an accumulator and an enrichment coefficient of <1 indicates an excluder plant. Typha angustifolia and Echhornia crassipus are accumulator plants. Heavy metals in decreasing sequence in T. angustifolia were found to be Ni>Pb>Fe>Zn>Cu in the root system, and in the shoot, accumulation of heavy metals was in the order of Fe>Ni>Pb> Zn>Cu. The TF value was found to be >1 in all cases except Mn, where concentration was found to be below detection limit.

Regarding Echhornia crassipus, concentrations in descending order of Fe>Pb>Zn>Ni>Cu were observed in the root system and Ni>Fe> Zn>Pb>Cu in the shoot system. The translocation factor was found to be >1 in all of the cases except Mn, where concentration was found to be below the detection limit.

## Discussion

Toxic metal contamination is increasing and has become a worldwide public health concern.^25^, ^26^ Manganese, Cu, Zn, Ni, Pb and Fe compounds cause a variety of adverse human health effects. Copper toxicity leads to retention of Cu in the kidney. Copper begins to deposit in the liver, disrupting the liver's ability to detoxify, further increasing Cu levels, and drastically affects the connective tissue, reproductive system, nervous system, adrenal function, and affects learning in newborn babies.^27^, ^28^

Zinc poisoning leads to gastrointestinal effects, such as abdominal pain, diarrhea and vomiting.^29^ In adverse cases, it may lead to sideroblastic anemia, hypochromic microcytic anemia and leukopenia that is primarily due to zinc-induced Cu deficiency. High levels of Zn are known to be cytotoxic and disrupt the homeostasis of other requisite elements.^30–32^

Nickel allergy can show up in the form of contact dermatitis, lung fibrosis, nasal and lung cancers, cardiovascular and kidney diseases.^33–37^ Lead poisoning can be either acute or chronic and lead to diseases pertaining to the central nervous system and gastrointestinal tract in children and adults.^38–40^ Acute exposure can result in headache, loss of appetite, hypertension, renal dysfunction, sleep apnea, vertigo, arthritis, abdominal pain, fatigue and hallucinations.^38^ Iron is a significant nutrient for most living creatures since it acts as a cofactor for many important enzymes and proteins. However, if proper shielding is not there, it can catalyze reactions involving formation of radicals that can damage the biomolecules, tissues, cells and the whole organism.^40^,^41^

Soil and water quality degradation and adverse effects on the human and animal health are largely attributed to industrial pollution, as they bioaccumulate in the food chain.^42^,^43^ Phytoremediation is an ecofriendly, low-cost, natural approach to clean up the environment.^44^,^45^ Metal accumulating plant species (Typha angustifolia and Echhornia crassipus) concentrate toxic and heavy metals such as Mn, Cu, Zn, Pb, Ni and Fe up to 100–1000 times, compared to excluder plants.^46^,^47^ Thus, phytoremediation is highly recommended for removal of toxic heavy metals from waterbodies and soil so that they do not enter the food chain and result in disease in humans and animals.

## Conclusions

The present study examined naturally growing vegetation in a domestic wastewater receiving lake, Laxmi Taal, in Jhansi, and indicated that parameters of wastewater in the lake were within the prescribed limit of water quality standard IS-2296 grade-D water. The bioconcentration factor study revealed that all of the values were approaching 1 or >1, thus T. angustifolia and E. crassipus are primary bioaccumulator plants.

Among the six heavy metals studied, Mn, Cu, Zn, Ni, Pb and Fe, the heavy metals TF value was found to be >1 in both T. angustifolia and E. crassipus. Heavy metals translocation from root to shoot was effective.

The heavy metal study revealed that accumulation of various metal ions by T. angustifolia and E. crassipus was higher in shoots than other parts of the plant. For all heavy metals, rhizofiltration and phytoextraction mechanisms have been found to be effective.

High levels of heavy metals have detrimental effects on the health of humans and animals. Monitoring of exposures and remedial measures are necessary to mitigate these effects. Phytoremediation has been found to be helpful in limiting the exposure of human beings and animals to the toxicity of water from lake Laxmi Taal, and in decreasing potential soil pollution, thus limiting the flow of these heavy metals further up the food chain. Phytoremediation by T. angustifolia and E. crassipus can be used effectively to help ensure waterbodies are free from toxic heavy metals dumped into waterways.

## References

[1] (2006). Water quality status of Yamuna River (1999 - 2005) [Internet].

[2] Newman MC, Unger MA (2002). Fundamentals of ecotoxicology.

[3] Reddy MV (2005). Restoration and management of tropical eutrophic lakes.

[4] Skoulikidis NT, Bertahas I, Koussouris T (1998). The environmental state of freshwater resources in Greece (rivers and lakes). Environ Geol [Internet].

[5] Clements WH, Newman MC (2002). Community ecotoxicology.

[6] Reddy MV, Kumar AV (2001). Effects of Ganesh-idol immersion on some water quality parameters of Hussainsagar Lake. Curr Sci.

[7] Vyas A, Bajpai A, Verma N (2008). Water quality improvement after shifting of idol immersion site: a case study of Upper Lake, Bhopal, India. Environ Monit Assess [Internet].

[8] Jasrotia S, Kansal A, Mehra A (2017). Performance of aquatic plant species for phytoremediation of arsenic-contaminated water. Appl Water Sci [Internet].

[9] Sumiahadi A, Acar R (2018). A review of phytoremediation technology: heavy metals uptake by plants. IOP Conf Ser Earth Environ Sci [Internet].

[10] Li J, Yu H, Luan Y (2015). Meta-analysis of the copper, zinc, and cadmium absorption capacities of aquatic plants in heavy metal-polluted water. Int J Environ Res Public Health [Internet].

[11] Coupel SJ, Sallami K, Ganjian E (2013). Phytoremediation of heavy metal contaminated soil using different plant species. Afr J Biotechnol [Internet].

[12] Otaru AJ, Ameh CU, Okafor JO, Odigure JO, Adbulkareem AS, Ibrahim S (2013). Study on the effectiveness of phytoremediation in the removal of heavy metals from soil using corn. Int J Comput Eng Res [Internet].

[13] Mojiri A, Azizi HA, Zahed MA, Aziz SQ, Selamat RB (2013). Phytoremediation of heavy metals from urban waste leachate by southern cattail (Typha domingensis). Int J Sci Res Environ Sci.

[14] Lu G, Wang B, Zhang C, Li S, Wen J, Lu G, Zhu C, Zhou Y (2018). Heavy metals contamination and accumulation in submerged macrophytes in an urban river in China. Int J Phytoremediation [Internet].

[15] Oh K, Cao T, Li T, Cheng H (2014). Study on application of phytoremediation technology in management and remediation of contaminated soils. J Clean Energy Technol [Internet].

[16] Arreghini S, de Cabo L, Serafini RJ, Fabrizio de Iorio A (2018). Shoot litter breakdown and zinc dynamics of an aquatic plant, Schoenoplectus californicus. Int J Phytoremediation [Internet].

[17] Liao SW, Chang WL (2004). Heavy metal phytoremediation by water hyacinth at constructed wetlands in Taiwan. J Aquat Plant Manage.

[18] Zaranyika MF, Ndapwadza T (1995). Uptake of Ni, Zn, Fe, Co, Cr, Pb, Cu and Cd by water hyacinth (Eichhornia crassipes) in Mukuvisi and Manyame rivers, Zimbabwe. J Environ Sci Health A [Internet].

[19] Singh D, Chhonkar PK, Pandey RN (1999). Soil plant water analysis: a method manual.

[20] Clescerl LS, Greenberg AE, Eaton AD (1998). Standard methods for the examination of water and wastewater.

[21] Horwitz W (2002). Metals and other elements in plants and pet foods (method No. 985.01). Official methods of analysis of AOAC International.

[22] Maiti SK, Jaiswal S (2008). Bioaccumulation and translocation of metals in the natural vegetation growing on fly ash lagoons: a field study from Santaldih thermal power plant, West Bengal, India. Environ Monit Assess [Internet].

[23] Kibria G (2004). Environmental update - dissolved oxygen: the facts. Outlet.

[24] Water Resources Systems Division, National Institute of Hydrology Jalvigyan Water quality requirement for different uses. http://117.252.14.242/rbis/india_information/water%20quality%20standards.htm.

[25] Nriagu JO (1990). Global metal pollution: poisoning the biosphere?. Environ Sci Policy Sustain Dev [Internet].

[26] Candelone JP, Hong S, Pellone C, Boutron CF (1995). Post-Industrial Revolution changes in large-scale atmospheric pollution of the northern hemisphere by heavy metals as documented in central Greenland snow and ice. J Geophys Res Atmospheres [Internet].

[27] Wilson L (2011). Copper toxicity syndrome.

[28] Ashish B, Neeti K, Himanshu K (2013). Copper toxicity: a comprehensive study. Res J Recent Sci.

[29] Gibson RS (1994). Zinc nutrition in developing countries. Nutr Res Rev [Internet].

[30] Markowitz M (2000). Lead poisoning. Pediatr Rev.

[31] Graham GA, Byron G, Norris RH (1986). Survival of Salmo gairdneri (rainbow trout) in the zinc polluted Molonglo River near Captains Flat, New South Wales, Australia. Bull Environ Contam Toxicol.

[32] Greger JL, Baligar P, Abernathy RP, Bennett OA, Peterson T (1978). Calcium, magnesium, phosphorus, copper, and manganese balance in adolescent females. Am J Clin Nutr [Internet].

[33] Uddin AN, Burns FJ, Rossman TG, Chen H, Kluz T, Costa M (2007). Dietary chromium and nickel enhance UV-carcinogenesis in skin of hairless mice. Toxicol Appl Pharmacol [Internet].

[34] Seilkop SK, Oller AR (2003). Respiratory cancer risks associated with low-level nickel exposure: an integrated assessment based on animal, epidemiological, and mechanistic data. Regul Toxicol Pharmacol [Internet].

[35] Gillette B (2008). Nickel named « allergen of the year », ACDS adds to list of substances warranting more attention. Dermatology Times.

[36] Viemann D, Schmidt M, Tenbrock K, Schmid S, Muller V, Klimmek K, Ludwig S, Roth J, Goebeler M (2007). The contact allergen nickel triggers a unique inflammatory and proangiogenic gene expression pattern via activation of NF-kappaB and hypoxiainducible factor-1alpha. J Immunol [Internet].

[37] Sivulka DJ (2005). Assessment of respiratory carcinogenicity associated with exposure to metallic nickel: A review. Regul Toxicol Pharmacol [Internet].

[38] Brochin R, Leone S, Phillips D, Shepard N, Zisa D, Angerio A (2008). The cellular effect of lead poisoning and its clinical picture. GU J Health Sci [Internet].

[39] Martin S, Griswold W (2009). Human health effects of heavy metals. Environ Sci Technol Briefs Citiz.

[40] Albretsen J (2006). The toxicity of iron, an essential element. Vet Med.

[41] Yoon J, Cao X, Zhou Q, Ma LQ (2006). Accumulation of Pb, Cu, and Zn in native plants growing on a contaminated Florida site. Sci Total Environ [Internet].

[42] Muchuweti M, Birkett JW, Chinyanga E, Zvauya R, Scrimshaw MD, Lester JN (2006). Heavy metal content of vegetables irrigated with mixtures of wastewater and sewage sludge in Zimbabwe: implications for human health. Agric Ecosyst Environ [Internet].

[43] Khan AG, Kuek C, Chaudhry TM, Khoo CS, Hayes WJ (2000). Role of plants, mycorrhizae and phytochelators in heavy metal contaminated land remediation. Chemosphere [Internet].

[44] Basta NT, McGowen SL (2004). Evaluation of chemical immobilization treatments for reducing heavy metal transport in a smelter-contaminated soil. Environ Pollut [Internet].

[45] Salt DE, Blaylock M, Kumar NP, Dushenkov V, Ensley BD, Chet I, Raskin I (1995). Phytoremediation: a novel strategy for the removal of toxic metals from the environment using plants. Biotechnol [Internet].

[46] Salt DE, Smith RD, Raskin I (1998). Phytoremediation. Annu Rev Plant Physiol Plant Mol Biol [Internet].

[47] Wang K, Zhang J, Zhu Z, Huang H, Li T, He Z, Yang X, Alva A (2012). Pig manure vermicompost (PMVC) can improve phytoremediation of Cd and PAHs co-contaminated soil by Sedum alfredii. J Soils Sediments [Internet].

